# Strangulated Inguinal Hernia at Nine Weeks of Age

**Published:** 1868-09-15

**Authors:** Curtis J. Fenn

**Affiliations:** 234 Thirty-first Street; Chicago


### STRANGULATED INGUINAL HERNIA AT NINE WEEKS OF AGE.

BY CURTIS J. FENN, M.D., CHICAGO.

THE little subject was born of young and healthy parents, and began immediately to thrive upon its mother's milk. It cried a good deal at times, and had occasional diarrhœa, but of easy control. It was finally taken to the country, in the vicinity of Blue Island, with benefit; on returning at the end of a fortnight it seemed to have more than ordinary promise. The mother carried it constantly on her lap, so that no direct injury could have happened to it. The night following its return it suffered with the colic, as the parents thought. Nothing gave it ease. Its cries became distressing, and continued with but slight intermissions. In the morning a tumor was discovered in the scrotum.

I first saw the case at 8, A.M. A fine, chubby boy. Its cries were pitiable in the extreme, between intervals of a few minutes sleep. It nursed; did not vomit; the abdomen was slightly tympanitic; the bowels had moved scantily morning and evening of the day before. The scrotum was œdematous and red; a tumor existing within the right side, oval shaped, about an inch in its long diameter, and extending along the inguinal canal. The right testicle was not perceived; over the most prominent part the swelling was a shade darker. An attempt to reduce its size by manipulation was unsuccessful; no impulse was felt over the external abdominal ring. Hot clothes were applied, and camphorated tincture of opium given in sufficient quantity to produce continued sleep.

Three hours later the hardness of the tumor was diminished, but on crying again its size was increased. Persistent efforts were now made for its reduction by means of *Chloroform* and pressure, with partial success; 12 M. there was a small evacuation of the bowels; 1 P.M., the size of the tumor had been reduced one-half. There was by this time occasional vomiting of natural matter from the stomach. Digital compression was continued through the whole afternoon. The tumor was further diminished in size, but became harder, the hardness extending up the line of the cord. The child lay profoundly sleeping the rest of the day. In the night its cries became incessant. *Paregoric* in large doses failed to quiet.

Early the second day prostration was marked. The shade of discoloration was deeper, and the tumefaction greater. It no longer took the breast. Green matters were sometimes vomited. The features were pale and sunken, and profound sleep had come again to the child's relief. 11 A.M. Prof. Gunn operated for the strangulation. Through an incision not more than an inch and a quarter long he found a deeply purple knuckle of intestine, filledwith hardened fæces. He opened the sack, slightly enlarged the internal ring, inserted it within the abdomen, and closed the wound with three delicate sutures. The testicle was now in its proper place, appearing natural. No blood had been lost. A light compress was closely fitted, and the child placed on its back and allowed quiet. It was comfortable during the afternoon. Three or four movements of the bowels followed during the night, discharges green. At the same time it became very restless again. *Paregoric* was given freely.

In the morning of the third day it was quietly sleeping. Its skin was hot and dry. The wound was pale. Milk and wine were given through the day, and at night one-sixteenth of a grain of sulphate of morphia; afterward repeated.

In the morning of the fourth day it lay perfectly quiet; took nourishment as before. The wound looked healthy. In the afternoon slight convulsions were excited during a thunder storm, and continued with little intermission for several hours. Its extremities became cold and pulseless, its respiration almost imperceptible. It afterward revived, then gradually sank, and, about noon of the fifth day, died without a struggle.

The case illustrates the danger of strangulation in congenital hernia; the exhaustion attendant upon suffering, and the fatal policy of delaying an operation when it is to become necessary.

234 Thirty-first Street, *August* 26*th*, 1868.

